# Endophytic fungi protect tomato and nightshade plants against *Tuta absoluta* (Lepidoptera: Gelechiidae) through a hidden friendship and cryptic battle

**DOI:** 10.1038/s41598-020-78898-8

**Published:** 2020-12-17

**Authors:** Ayaovi Agbessenou, Komivi S. Akutse, Abdullahi A. Yusuf, Sunday Ekesi, Sevgan Subramanian, Fathiya M. Khamis

**Affiliations:** 1 International Centre of Insect Physiology and Ecology (icipe), P.O. Box 30772-00100, Nairobi, Kenya; 2 Department of Zoology and Entomology, University of Pretoria, Private Bag X20, Hatfield, 0028 South Africa; 3 Forestry and Agricultural Biotechnology Institute (FABI), University of Pretoria, Private Bag X20, Hatfield, 0028 South Africa

**Keywords:** Biological techniques, Ecology, Microbiology

## Abstract

Endophytic fungi live within plant tissues without causing any harm to the host, promote its growth, and induce systemic resistance against pests and diseases. To mitigate the challenging concealed feeding behavior of immature stages of *Tuta absoluta* in both tomato (*Solanum lycopersicum*) and nightshade (*Solanum scabrum*) host plants, 15 fungal isolates were assessed for their endophytic and insecticidal properties. Twelve isolates were endophytic to both host plants with varied colonization rates. Host plants endophytically-colonized by *Trichoderma asperellum* M2RT4, *Beauveria bassiana* ICIPE 706 and *Hypocrea lixii* F3ST1 outperformed all the other isolates in reducing significantly the number of eggs laid, mines developed, pupae formed and adults emerged. Furthermore, the survival of exposed adults and F1 progeny was significantly reduced by *Trichoderma* sp. F2L41 and *B. bassiana* isolates ICIPE 35(4) and ICIPE 35(15) compared to other isolates. The results indicate that *T. asperellum* M2RT4, *B. bassiana* ICIPE 706 and *H. lixii* F3ST1 have high potential to be developed as endophytic-fungal-based biopesticide for the management of *T. absoluta*.

## Introduction

Vegetable production is one of the most viable horticultural sub-sector in Africa and is considered an important route out of poverty for smallholder farmers^1^. Tomato (*Solanum lycopersicum* L.; Solanaceae) is one of the most promising vegetable for horticultural expansion in Africa but the crop is experiencing significant losses due to abiotic and biotic stressors threatening the livelihoods of millions of smallholder farmers^2^. Among the biotic factors, the invasive tomato leafminer *Tuta absoluta* (Meyrick) (Lepidoptera: Gelechiidae), which originated from South America and spread as far as Europe^3^ has emerged as one of the most important devastating pest of tomato during the last decade, contributing to increasing risk of malnutrition and food insecurity in Africa. In addition to tomato crop, the pest also attacks various cultivated and wild plants within the Solanaceae family such as pepper, *Capsicum annuum* L.; eggplant, *Solanum melongena* L.; tobacco, *Nicotiana tabacum* L.; potato, *Solanum tuberosum* L. and black nightshade, *Solanum nigrum* L.^4^. In Kenya, both tomato and nightshade crops are the most preferred hosts for the tomato leafminer with high infestation causing up to 100% yield losses on tomato^4^. Estimates of the economic losses due to this pest reaches as high as US$ 59.3 million annually^5^. Ovipositing female lays eggs on the upper surface of tomato leaves which hatch after four to five days. Neonate larvae penetrate the leaf and feed on the mesophyll resulting in the production of thin and irregular mines on the leaf surface compromising the photosynthetic activity of the plant that negatively affect crop productivity or yield^6^. Mature larvae bore into the tomato leaves, fruits and flowers, spending most of their lifespan inside the crop than outside^7^. This concealed feeding behavior allows the pest to escape from most of the synthetic insecticides currently being applied hindering management of the pest. The resultant high use of synthetic pesticides causes significant short- and long-term adverse environmental and human health effects and increased resistance development in *T. absoluta*^8^; emphasizing therefore the need to promote environmentally-friendly control methods to curtail these problems. As a viable alternative to the use of synthetic insecticides, the development of biological control approaches using entomopathogenic fungi has shown promising results as they cause high mortality to insect pests of economic importance^9–12^. Akutse et al.^13^ reported the potential of fungal pathogens to control the pest and subsequently identified three *Metarhizium anisopliae* (Metschnikoff) Sorokin strains (ICIPE 18, ICIPE 20 and ICIPE 655) as candidate biopesticides causing mortality of 95.0, 87.5 and 86.25%, respectively against the adult stage of the pest. Entomopathogenic fungi have been traditionally used to control insect pests mostly through inundative application^14^. But recent studies have begun to examine their activity as plant endophytes to systemically protect plants against herbivorous insect pests^15^ and are therefore best suited to target the cryptic stages of *T. absoluta* such as larvae and pupae^16^.

Endophytic fungi are symptomless microbial organisms that live within host plant tissues either naturally or through artificial inoculation without causing any outward harm to the host^17^. Some of the advantages of using endophytic fungi compared to other biocontrol agents reside in the fact that, they are less exposed to the effect of environmental stresses and require little inoculum for its systemic delivery within the host plant tissues^18^. In some cases, these ubiquitous fungi play an important role as plant growth promoters participating therefore in the acquisition of nutrients by the plants^19^. Although several inoculation methods have been reported to be effective in delivering the inoculum at the target site, insecticidal seed treatment has been termed as the most convenient, very safe and cost-effective inoculation method for successful endophytic colonization of many crop plants^20^. Consequently, using this delivery technique, host-adapted endophytes have been successfully established in tomatoes^21^, Faba bean^22,23^, maize^24^ and cotton^21^. Upon plant colonization, endophytic fungi help their host plants to perform better under stressful environmental conditions (drought) and withstand biotic stressors (pathogens and herbivores) through the induction of local or systemic resistance, antibiosis, phytohormones production and the stimulation of plant secondary metabolites^25,26^.

In an attempt to improve the management of the pea leafminer *Liriomyza huidobrensis* (Blanchard) (Diptera: Agromyzidae), Akutse et al.^23^ and Gathage et al.^27^ reported that through seed inoculation, endophytic fungi could successfully colonize Faba beans plant tissues and cause significant suppression of the pest. A similar study by Muvea et al.^28^ reported on the establishment of endophytic fungi within onion plant and the ability of these microorganisms in reducing the population of onion thrips, *Thrips tabaci* Lindeman (Thysanoptera: Thripidae) on inoculated plants. Similarly, tomato seeds pre-treated with the endophytic fungi *Beauveria bassiana* (Balsamo-Criv.) Vuillemin reduced larval performance of *Helicoverpa zea* (Boddie) (Lepidoptera: Noctuidae)^29^. Recently, Klieber and Reineke^30^ revealed that endophytic fungi inoculated in tomato plants mediated systemic resistance against *T. absoluta* and played a significant role in reducing feeding activity of the immature stage of the pest. This pest control strategy has added a new dimension to the use of fungal entomopathogens against cryptic insect pests whose life cycle limits the effectiveness of chemical insecticides and other control methods^18,31^. Therefore, to tackle the concealed feeding behavior of the larval stage of *T. absoluta*, the objective of this research was to assess the endophytic properties of fifteen fungal isolates on both tomato and nightshade plants and evaluate their insecticidal activity or pathogenicity with their ability to induce systemic resistance against the pest with the aim to use the potent fungal endophytic-based biopesticide as a component of a Tuta-IPM.

## Results

### Endophytic colonization of tomato and nightshade by fungal isolates

**Figure 1:**
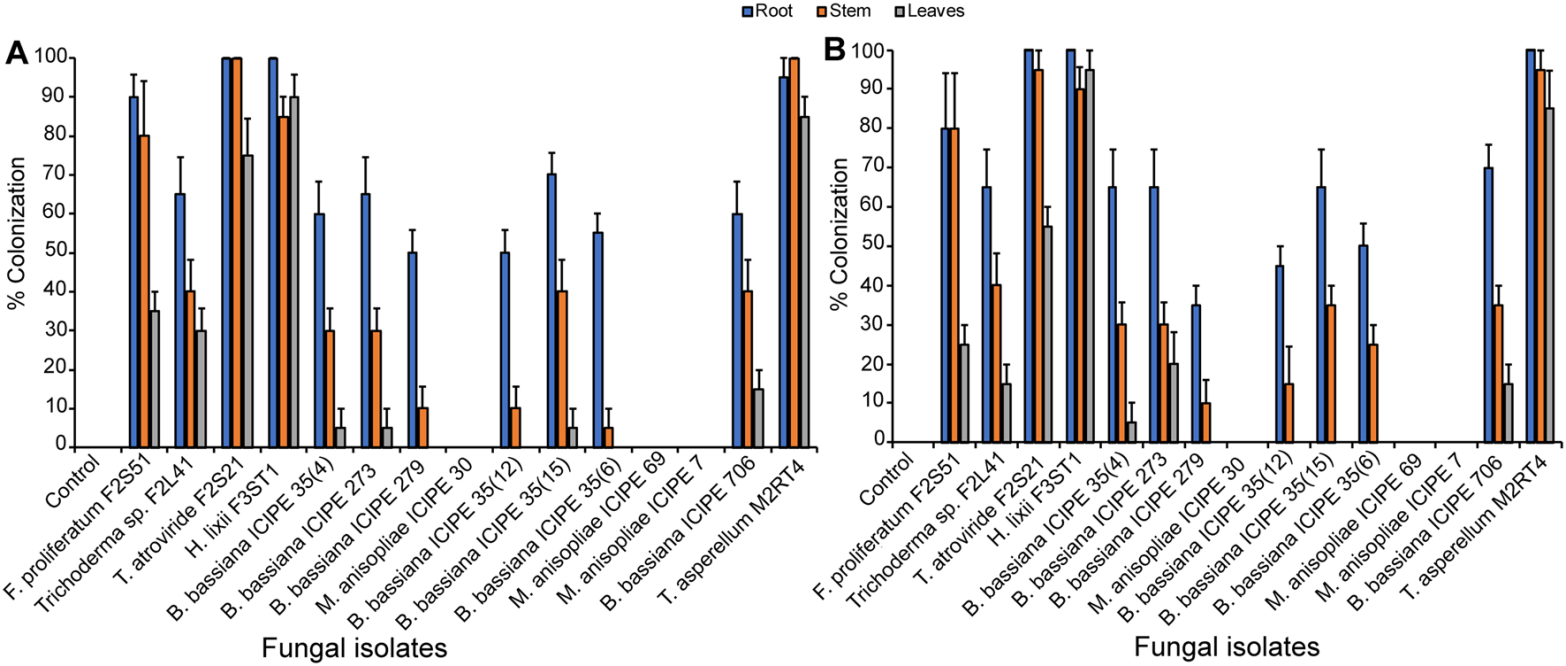
Endophytic colonization of tomato *Solanum lycopersicum* (**A**) and nightshade *Solanum scabrum* (**B**) host plant parts by 15 fungal isolates at 4–5 weeks post-inoculation. Bar chart represents means ± SE (standard error) at 95% CI (*P* < 0.05; n = 4).

The results of viability tests showed that conidia germination of the different fungal isolates used in this study exceeded 90% after 18 h of incubation. Endophytic colonization rate was determined by the recovery of the inoculated fungal strains from the roots, stems and leaves, respectively. The 15 fungal isolates differed markedly in their ability to colonize both tomato and nightshade plants. Irrespective of the host plants, *M*. *anisopliae* isolates ICIPE 30, ICIPE 69 and ICIPE 7 failed to colonize the various plant parts while the remaining 12 isolates were successfully recovered from tomato and nightshade host plant parts (Fig. 1A,B). However, colonization of the different plant tissues (roots, stems and leaves) varied depending on fungal isolates and host plants. For example, isolates of *F. proliferatum* F2S51, *Trichoderma* sp. F2L41, *T. atroviride* F2S21, *H. lixii* F3ST1, *B. bassiana* ICIPE 35(4), ICIPE 273, ICIPE 706 and *T. asperellum* M2RT4 colonized roots, stems and leaves of both host plants. *Beauveria bassiana* ICIPE 35(15) colonized the roots, stems, and leaves of tomato plants while it colonized only the roots and stems of nightshade (Fig. 1A,B). It is worth noting that; *B. bassiana* ICIPE 35(12), ICIPE 35(6) and ICIPE 279 colonized only roots and stems of both host plants. In addition, *H. lixii* F3ST1 and *T. asperellum* M2RT4 colonized more than 85% of all the plant tissues of both host plants while *B. bassiana* ICIPE 706 colonized 60, 40 and 15% of roots, stems and leaves of tomato, respectively (Fig. 1A); and 70, 35 and 15% of roots, stems and leaves of nightshade, respectively (Fig. 1B). *Trichoderma atroviride* F2S21 successfully colonized 100, 100 and 75% of roots, stems and leaves of tomato plant respectively, and 100, 95 and 55% of roots, stems and leaves in nightshade, respectively (Fig. 1A,B). Significant differences in colonization by isolates were observed on roots (χ^2^ = 112.31, df = 11, *P* < 0.0001), stems (χ^2^ = 204.36, df = 11, *P* < 0.0001) and leaves (χ^2^ = 279.74, df = 11, *P* < 0.0001) of tomato (Fig. 1A). Similarly, significant differences were observed in colonization levels of plant parts of nightshade: roots (χ^2^ = 114.17, df = 11, *P* < 0.0001), stems (χ^2^ = 131.89, df = 11, *P* < 0.0001) and leaves (χ^2^ = 297.73, df = 11, *P* < 0.0001) (Fig. 1B).

### Effect of endophytically-colonized tomato and nightshade host plants on survival of adult *Tuta absoluta*

**Figure 2:**
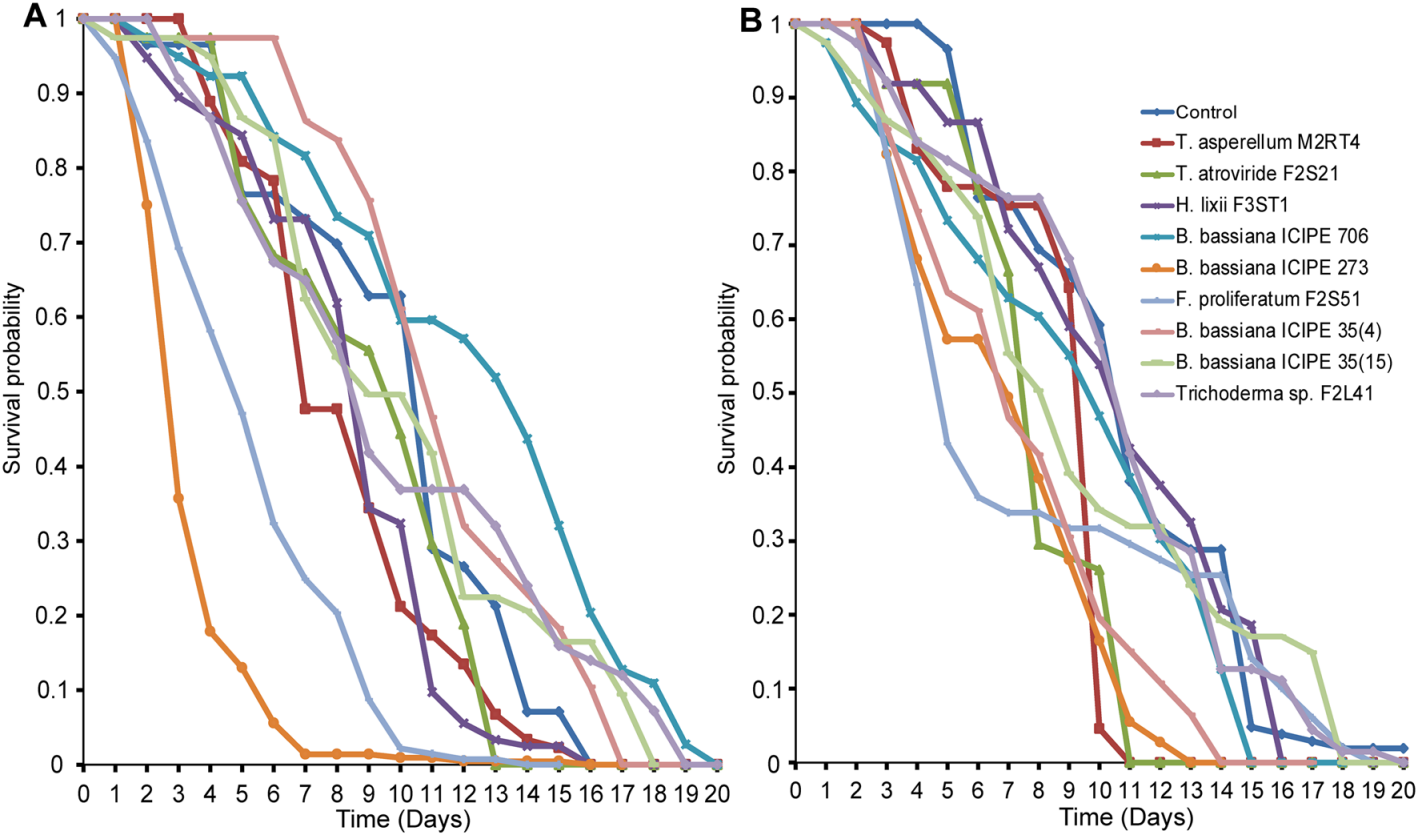
Effect of endophytically-colonized host plants by fungal isolates on survival of adult *Tuta absoluta*: (**A**) Kaplan–Meier survival curves of *Tuta absoluta* adults exposed to endophytically-colonized tomato plants, (**B**) Kaplan–Meier survival curves of *Tuta absoluta* adults exposed to endophytically-colonized nightshade plants (*P* < 0.05, n = 4).

The survival of *T. absoluta* adults exposed to endophytically-colonized tomato plants varied significantly among the treatments (Proximate log rank test, χ^2^ = 168.5, df = 9, *P* < 0.0001). For example, at day 5 post-exposure, mean adult survival was 28.21% with *B*. *bassiana* ICIPE 273 and 32.69% with *F. proliferatum* F2S51 compared to 52.28% in the control (Fig. 2A). At day 10 post-exposure, mean adult survival ranged between 9.2 and 26.40% including the control, except for *B*. *bassiana* ICIPE 706 (30.80%). At day 15 post-exposure, the survival was less than 10% including the control. At day 20 post-exposure, no survival was observed in all the treatments including the control (Fig. 2A). Similarly, there was a significant difference in the survival of *T. absoluta* adults exposed to endophytically-colonized nightshade plants (Proximate log rank test, χ^2^ = 82.79, df = 9, *P* < 0.0001) compared to the control. At day 5 post-exposure, mean adult survival was between 39 and 54% including the control (Fig. 2B). At day 10 post-exposure, mean adult survival ranged between 10 and 26.6% including the control. At day 15 post-exposure, no survival was observed in *T. asperellum* M2RT4 while mean adult survival was below 10% in all the treatments including the control (Fig. 2B).

### Effect of endophytically-colonized tomato and nightshade host plants on oviposition and leafmining of *Tuta absoluta*

**Figure 3:**
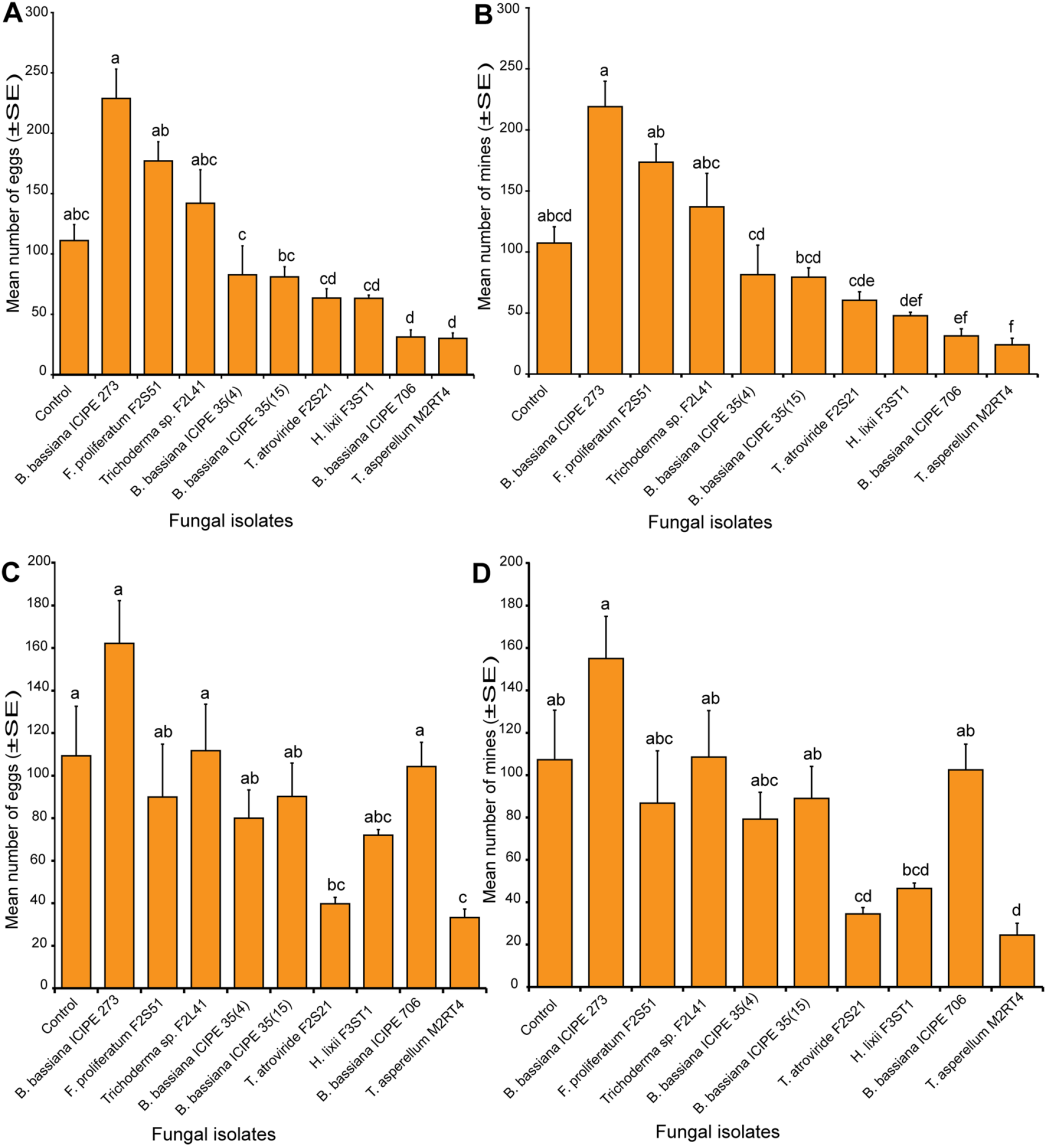
Effect of endophytically-colonized host plants by fungal isolates on oviposition and leafmining of *Tuta absoluta* at 48 h post-exposure. (**A**) Bar chart showing mean number (± SE) of *Tuta absoluta* eggs laid on endophytically-colonized tomato plants. (**B**) Bar chart showing mean number (± SE) of mines produced by *Tuta absoluta* on endophytically-colonized tomato plants. (**C**) Bar chart showing mean number (± SE) of *Tuta absoluta* eggs laid on endophytically-colonized nightshade plants. (**D**) Bar chart showing mean number (± SE) of mines produced by *Tuta absoluta* on endophytically-colonized nightshade plants. Means followed by a different lowercase letters are significantly different (*P* < 0.05; n = 4; Tukey’s HSD test).

The number of eggs laid on endophytically-colonized tomato plants varied significantly among the treatments (χ^2^ = 208.92, df = 9, *P* < 0.0001) (Fig. 3A). For instance, *T. asperellum* M2RT4 endophytically-colonized tomato plants recorded the lowest number of eggs (30.0 ± 4.51 eggs), followed by *B. bassiana* ICIPE 706 (31.25 ± 5.88 eggs), *H. lixii* F3ST1 with 63.25 ± 2.66 eggs and *T. atroviride* F2S21 with 63.5 ± 7.63 eggs, compared to 111.0 ± 13.32 eggs in the control (Fig. 3A). However, the highest number of eggs was recorded on *B. bassiana* ICIPE 273 (228.75 ± 24.36 eggs), followed by *F. proliferatum* F2S51 (177.0 ± 15.96 eggs), *Trichoderma* sp. F2L41 with 142.0 ± 27.67 eggs and the control (111.0 ± 13.32 eggs) (Fig. 3A). Upon egg hatching, *T*. *asperellum* M2RT4-endophytically-colonized tomato plants recorded the lowest number of mines (24.0 ± 5.4 mines) while *B. bassiana* ICIPE 273 recorded the highest number of mines (219.0 ± 20.92 mines) followed by *F. proliferatum* F2S51 (173.5 ± 15 mines), *Trichoderma* sp. F2L41 with 137.0 ± 24.47 mines, compared to 107.33 ± 13.32 mines in the control (χ^2^ = 216.4, df = 9, *P* < 0.0001) (Fig. 3B).

Similarly, endophytically-colonized nightshade plants had a significant effect on the oviposition of *T. absoluta* (χ^2^ = 91.73, df = 9, *P* < 0.0001) (Fig. 3C). Among the fungal isolates, the lowest number of eggs was laid on *T*. *asperellum* M2RT4 endophytically-colonized nightshade plants (33.25 ± 3.97 eggs) compared to 109.33 ± 23.31 eggs in the control (Fig. 3C). However, the highest number of eggs (162.25 ± 20.01 eggs) was recorded on *B. bassiana* ICIPE 273, followed by *Trichoderma* sp. F2L41 with 111.75 ± 21.85 eggs and *B. bassiana* ICIPE 706 (104.25 ± 11.38 eggs), compared to 109.33 ± 23.31 eggs in the control (Fig. 3C). Subsequently, following egg hatchability, the lowest number of mines (24.5 ± 5.55 mines) was recorded on *T*. *asperellum* M2RT4 endophytically-colonized nightshade plants while the highest was recorded on *B. bassiana* ICIPE 273 (155.0 ± 19.94 mines) followed by *Trichoderma* sp. F2L41 (108.5 ± 22.02 mines) and the control (107.33 ± 23.31 mines) (χ^2^ = 110.95, df = 9, *P* < 0.0001) (Fig. 3D).

### Effect of endophytically-colonized tomato and nightshade host plants on *Tuta absoluta* pupation and adult emergence

**Figure 4:**
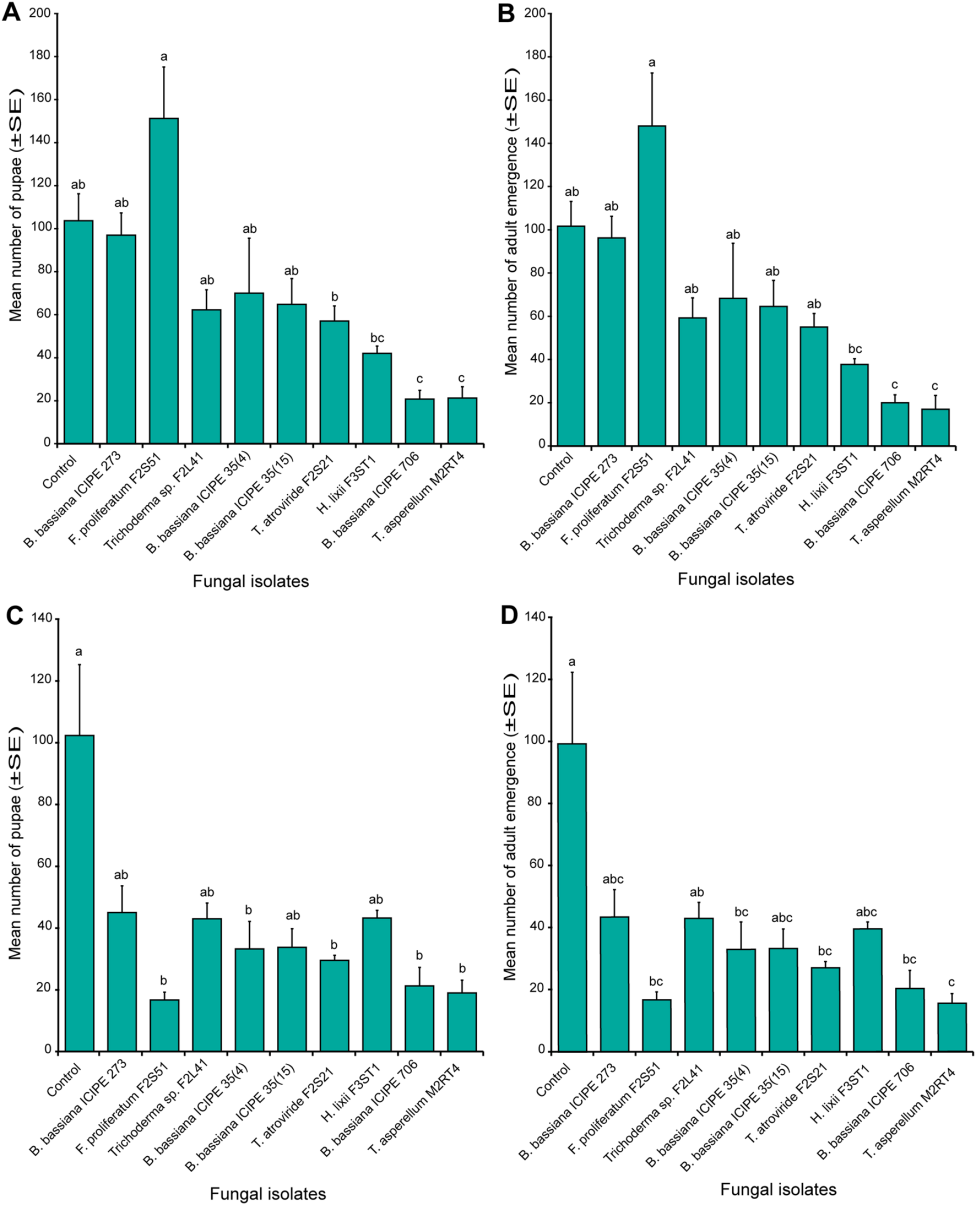
Effect of endophytically-colonized host plants by fungal isolates on *Tuta absoluta* pupation and adult emergence. (**A**) Bar chart showing mean number (± SE) of *Tuta absoluta* pupae produced on endophytically-colonized tomato plants. (**B**) Bar chart showing mean number of *Tuta absoluta* adults emerging from endophytically-colonized tomato plants. (**C**) Bar chart showing mean number (± SE) of *Tuta absoluta* pupae produced on endophytically-colonized nightshade plants. (**D**) Bar chart showing mean number of *Tuta absoluta* adults emerging from endophytically-colonized nightshade plants. Means followed by a different lowercase letters are significantly different (*P* < 0.05; n = 4; Tukey’s HSD test).

The pupation of *T. absoluta* larvae that survived was significantly affected (χ^2^ = 131.45, df = 9, *P* < 0.0001) by the endophytically-colonized tomato plants (Fig. 4A). In endophytically-colonized tomato plants, fewer *T. absoluta* pupae (20.75 ± 4.05 pupae) were produced in *B. bassiana* ICIPE 706 followed by *T*. *asperellum* M2RT4 (21.25 ± 5.22 pupae) which were significantly different from *F. proliferatum* F2S51 (151.25 ± 23.92 pupae) and the control (103.67 ± 12.55 pupae) (Fig. 4A). Further, *T. absoluta* adult emergence varied significantly among the fungal isolates (χ^2^ = 58.34, df = 9, *P* < 0.01), where the highest number of moths (148.0 ± 24.57) emerged from *F. proliferatum* F2S51 endophytically-colonized tomato plants, followed by the control 101.67 ± 11.46 while the lowest number (17.0 ± 6.34 moths) was recorded on *T. asperellum* M2RT4 endophytically-colonized tomato plants (Fig. 4B).

Pupal formation was significantly different among the treatments (χ^2^ = 90.95, df = 9, *P* < 0.0001) where the highest number was obtained in the control (102.33 ± 22.93 pupae) and the lowest (19 ± 4.12 pupae) was recorded in *T. asperellum* M2RT4 endophytically-colonized nightshade plants (Fig. 4C). Further, the number of adults that emerged from the control (99.33 ± 22.98 moths) was significantly higher than the lowest number (15.5 ± 3.2 moths) that was obtained in *T. asperellum* M2RT4 endophytically-colonized nightshade plants (χ^2^ = 44.99, df = 9, *P* < 0.0001) (Fig. 4D).

### Effect of endophytically-colonized tomato and nightshade host plants on *Tuta absoluta* F1 progenies survival

**Figure 5:**
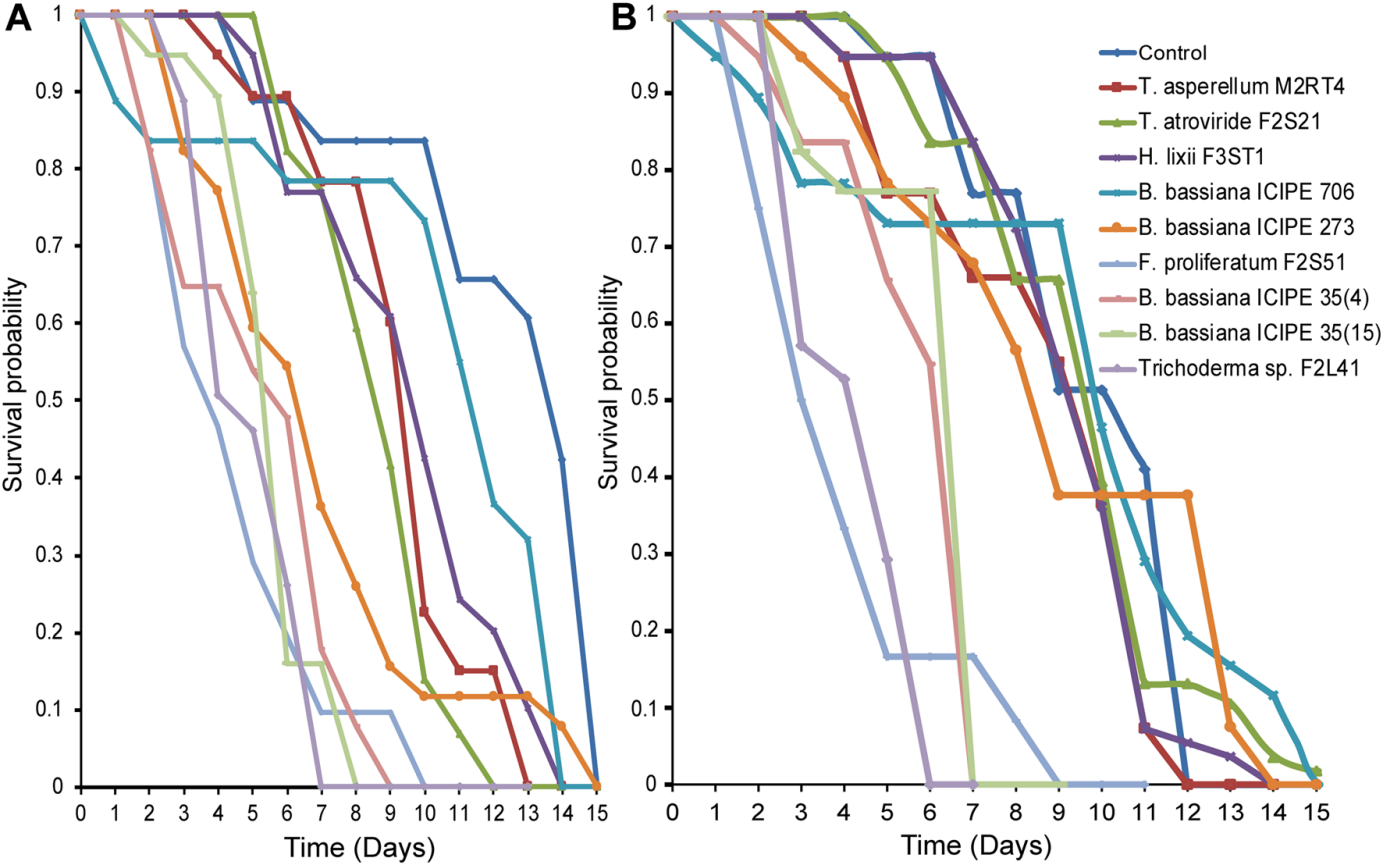
Effect of endophytically-colonized host plants by fungal isolates on *Tuta absoluta* F1 progenies survival. (**A**) Kaplan–Meier survival curves of *Tuta absoluta* F1 progenies survival emerging from endophytically-colonized tomato plants. (**B**) Kaplan–Meier survival curves of *Tuta absoluta* F1 progenies survival emerging from endophytically-colonized nightshade plants (*P* < 0.05, n = 4).

The median survival time of F1 progenies from the endophytically-colonized tomato plants varied significantly among the treatments (Proximate log rank test, χ^2^ = 180.7, df = 9, *P* < 0.0001) (Fig. 5A). At day 5 post emergence, mean survival was between 15.6 and 24% in *Trichoderma* sp. F2L41, *B. bassiana* ICIPE 35(4), *B. bassiana* ICIPE 35(15) and *F. proliferatum* F2S51 compared to 58.16% in the control (Fig. 5A). At day 10 post emergence, there was no survival in *Trichoderma* sp. F2L41, *B. bassiana* ICIPE 35(4), *B. bassiana* ICIPE 35(15) while it was between 7 and 28% in other treatments including the control (Fig. 5A). Similarly, the survival of F1 progeny from endophytically-colonized nightshade plants revealed a significant difference among treatments (Proximate log rank test, χ^2^ = 128.9, df = 9, *P* < 0.0001) (Fig. 5B). At day 5 post emergence, mean survival was between 17 and 29% in *Trichoderma* sp. F2L41, *B. bassiana* ICIPE 35(4), *B. bassiana* ICIPE 35(15) and *F. proliferatum* F2S51 compared to 51.23% in the control (Fig. 5B). At day 10 post emergence, there was no survival in *Trichoderma* sp. F2L41, *B. bassiana* ICIPE 35(4), *B. bassiana* ICIPE 35(15) and *F. proliferatum* F2S51 while it ranged between 11 and 19% in other treatments including the control (Fig. 5B).

## Discussion

This study demonstrated successful endophytic colonization and establishment of some fungal isolates in tomato and nightshade host plants by negatively affecting *T. absoluta* through significant reduction of the pest oviposition capacity, leafmining, pupal formation, adult emergence and survival. *Trichoderma asperellum* M2RT4, *B. bassiana* ICIPE 706 and *H. lixii* F3ST1-endophytically-colonized host plants outperformed all the other endophytes in affecting all the life-history parameters of the pest and could therefore contribute to its suppression in tomato and other solanaceous crops.

Among the 15 fungal isolates tested, 12 were endophytic to both host plants with varying colonization rates while *M. anisopliae* isolates failed to colonize both host plants. Irrespective of the host plant, fungal isolates belonging to the genera *Fusarium* (*F. proliferatum* F2S51), *Trichoderma* (*T. asperellum* M2RT4, *T. atroviride* F2S21 and *Trichoderma* sp. F2L41) and *Hypocrea* (*H. lixii* F3ST1) have demonstrated high colonization rates of all tomato and nightshade plant tissues. These fungal isolates except for *Trichoderma* sp. F2L41 had a similar *in planta* colonization pattern in onion through seed inoculation as previously reported by Muvea et al.^28^. Mutune et al.^32^ also reported the potential of *T. asperellum* M2RT4, *T. atroviride* F2S21 and *H. lixii* F3ST1 to endophytically-colonize different parts of the common bean plant *Phaseolus vulgaris* L. (Fabaceae). This implies that the recovery of endophytic fungi from plant tissues (leaves, stems and roots) after seed inoculation is an indication of their ascending movement within the plant^33^. Previously, such systemic spread of endophytes within the plant has been reported to occur in several crops such as maize^34^, *Vicia faba* and *P. vulgaris*^23,35^, tomatoes^29^, bananas^36^ and coffee^37^. Some endophytic fungi have also been reported to display a differential ability to colonize and multiply in the root cortex of different plant species while others establish in the whole plant tissues^38^. Recently, a survey conducted on the prevalence and distribution of fungal root endophytes occurring in tomato crop in Kenya by Bogner et al.^39^ found that the most prevalent endophytic fungi associated with tomato roots were members of *Fusarium* and *Trichoderma* genera. This confirms observations of Hardoim et al.^40^ who reported that members of these two genera have the potential to colonize a wide range of hosts, suggesting their great metabolic and physiological adaptability.

In contrast to the successfully high colonization rates of plant tissues by *Fusarium*, *Trichoderma* and *Hypocrea*, the level of colonization of *B. bassiana* isolates varied according to the various plant tissues with low colonization rate found in the leaves. A probable explanation of the low recovery of *B. bassiana* from the aerial tissues could be due to the speed of colonization (inoculum migration) or the presence of physical barriers in the leaf which prevent the fungus from penetrating the epidermis which may contain some substances inimical to the growth of the fungus^33,41^. However, there are several research evidence that reported the ability of *B. bassiana* to colonize a wide range of plants belonging to the monocot and dicot groups including banana^36^, *V. faba*^42^, opium^43^, maize^44^, cassava^45^, tomato^29^ and coffee^33^. Nonetheless, this underscores the lack of host specificity expressed by this fungal species in both host seedlings^46^. In this study, the highest recovery of *B. bassiana* was from the roots of the plants which indicates that through seed inoculation this strain has gained access to the cells of the plant. This confirms the observation that many endophytic fungi originate from the rhizosphere microbiota, an environment which attracts microorganisms better due to the presence of root exudates and rhizodeposits^47^. On the other hand, Behie et al.^15^ reported that endophytic fungi may display preferential tissue colonization within their host plants owing to many factors, including plant tissue type, plant genotype, microbial taxon and strain type^40^. Even though *M. anisopliae* isolates ICIPE 7, ICIPE 30 and ICIPE 69 were reported to be pathogenic to several arthropod pests of economic importance^48^, their failure in colonizing both host plants indicates their inability to establish themselves in living plant tissues of tomato and nightshade. Similar results have been reported on other host plants such as French bean and Faba bean^23,32^. Additionally, perhaps not all insect-pathogenic fungi have the ability to establish themselves as endophytes in living plant tissues^49^. However, numerous studies have documented the ability of *Metarhizium* spp. to colonize plant roots providing multiple benefits to their host plants^45,50,51^.

In general, our results reveal that exposure of both endophytically-colonized host plants to ovipositing *T. absoluta* females has resulted in a significant reduction in the number of eggs laid on the inoculated plant compared to the control. Among the most potent endophytic fungal isolates, we found that *T. asperellum* M2RT4, *B. bassiana* ICIPE 706 and *H. lixii* F3ST1 significantly reduced oviposition of the pest. For instance, Muvea et al.^52^ demonstrated a sixfold reduction in oviposition of onion thrips on plants endophytically-colonized by *H. lixii* F3ST1 compared to endophyte-free plants. Also, Akutse et al.^23^ reported that Faba bean endophytically-colonized by *H. lixii* had significant effect on the egg-laying capacity of the pea leafminer *L*. *huidobrensis*. It is worth noting that the female's choice to reduce egg production could be due to the absence of favorable conditions that would compromise the survival of the progeny^53^. Furthermore, *T. asperellum* M2RT4 negatively affected leafmining activity as well as pupation and adult emergence. When the hatching larvae feed on inoculated tissue, it generally results in a decreased fitness of the herbivore^54^. This corroborates with Akutse et al.^23^ who reported that endophytic fungi provide systemic protection against the pea leafminer *L*. *huidobrensis* and deterrent effects on life-history parameters of the pest. In addition, several studies have also reported insecticidal activities of endophytic fungi against insects feeding on endophytically-colonized plants through antibiosis or feeding deterrence, suggesting that immature larvae were probably affected through the secretion of toxic compounds *in planta*^30,45,55–57^. The inhibition of the larval performance due to the presence of *Trichoderma* spp. within the host plants has previously been reported^58^. The systemic activity of this fungal isolate as one of the most potent endophytic fungal strain controlling *T. absoluta* was not surprising, since similar effects have been reported in previous studies by Akello and Sikora^22^ and Muvea et al.^28^ on aphids and thrips population, respectively. The latter indicated that onion thrips feeding on onion plants inoculated by *Trichoderma* spp. performed worse and few immature stages reached the adult stage compared to the control. This suggests that *T. asperellum* M2RT4 possesses specific properties that trigger plant resistance which results in significant reduction of insect herbivory^59^. Similarly, Coppola et al.^60^ reported an enhancement of the indirect defense barriers against the aphid *Macrosiphum euphorbiae* (Hemiptera: Aphididae) feeding on tomato plants colonized by *T. harzianum* T22.

On the other hand, we found that *B. bassiana* fungal isolates (ICIPE 706 and ICIPE 273) reduced leafmining activity as well as pupation although showing low level *in planta* colonization pattern. However, *B. bassiana* isolate ICIPE 706 had the highest negative impact on the pest oviposition, pupation and adult emergence in both host plants, while it reduced significantly the mines formation only in tomato. Since *T. absoluta* larvae continue to feed on inoculated plants after egg hatching due to their cryptic nature, the amount and quality of host diet could significantly affect the feeding behavior of the leafmining larvae. It is therefore possible that this low colonization level was sufficient for the plants to initiate a defense reaction^61^. Klieber and Reineke^30^ reported that *T. absoluta* larvae experienced detrimental effects when feeding on tomato leaves infected with *B. bassiana*. Lewis et al.^62^ also demonstrated that when *B. bassiana* remains in the maize plant as endophyte, it provides a season-long management of the European corn borer, *Ostrinia nubilalis* (Hübner) (Lepidoptera: Crambidae) through the reduction of the larval activity of the pest. Qayyum et al.^63^ reported that endophytic colonization of *B*. *bassiana* has potential as an effective strategy to control *Helicoverpa armigera* (Hübner) (Lepidoptera: Noctuidae) in tomatoes.

Not all the fungal isolates tested in this study were able to deter the oviposition behavior against *T. absoluta*. Of the tested isolates, three (*B. bassiana* ICIPE 273, *F. proliferatum* F2S51 and *Trichoderma* sp. F2L41) recorded high number of eggs compared to other treatments and the control (endophyte-free tomato and nightshade plants). Jensen et al.^42^ also found an increased fecundity of the second generation of *Aphis fabae* on *V. faba* plants following seed and leaf inoculation with *B. bassiana*. The authors further speculated that *B*. *bassiana* is responsible for the improvement of the quality of the host plant which have led the insects to increase the number of eggs laid on the inoculated plants. Similarly, Jallow et al.^64^ examined in tomato the systemic effects of the endophytic fungus *Acremonium strictum* on the oviposition behavior of the polyphagous moth *Helicoverpa armigera* (Hübner). The authors reported that strains of *H. armigera* moths oviposited more eggs on leaves of *A. strictum*-inoculated plants as compared to endophyte-free plants. Later, Jaber and Vidal^65^ suggested that the increased oviposition preference of *H. armigera* moths to inoculated plants might be an evolutionary adaptation to the host plant. Although we have not investigated/established the mechanism by which these three isolates (*B. bassiana* ICIPE 273, *F. proliferatum* F2S51 and *Trichoderma* sp. F2L41) increased the attractiveness to the two host plants for egg-laying in *T. absoluta*, our results suggest that secondary metabolites or microbial volatile organic compounds produced by these endophytes or the interaction of the plants with the fungi may play a role in influencing the host selection of *T. absoluta* for oviposition^66^. The difference in the number of eggs laid on the several inoculated plants is suggestive of chemical and/or molecular mechanism(s) mediating interaction between the endophytes, insect and its host plants, calling for further studies.

The results reported here showed that females exposed to both tomato and nightshade intact plants lived less than 20 days. This finding is in agreement with Silva et al.^67^ who reported that females *T. absoluta* had a lifespan less than 20 days. However, our result is in contrast with Pereyra and Sanchez^68^ who reported that the survival of *T. absoluta* individuals could be extended until day 45 and remained high most of the lifetime but start decreasing to 50% at day 25. These variations might be due to the experimental conditions or the food source provided to the emerged adults during the survival bioassays. Further, we found a rapid decline in the survival rates of *T. absoluta* F1 progenies that emerged from larvae that fed on *Trichoderma* sp. F2L41, *B. bassiana* ICIPE 35(4), *B. bassiana* ICIPE 35(15) endophytically-colonized host plants. Our results concur with Dash et al.^69^ who also found a reduction of the survival of adult spider mites whose larvae fed on endophytically-colonized bean plants. Akello et al.^70^ reported an antagonistic activity mediated by the endophytic fungus *B. bassiana* towards the banana weevil adult, *Cosmopolites sordidus* (Coleoptera: Curculionidae). However, we did not record any sign of fungal infection on the dead insects which suggests that a probable mechanism of systemic resistance or feeding deterrence would be the factor responsible for the adverse effect of the inoculated plants on adult survivorship. Such deterrence exhibited by inoculated plants is related to the production of secondary metabolites by some fungi which may be an interesting exploitable feature for their sustainable use against agricultural insect pests of economic importance^71^.

## Conclusion

In this study, we have identified *T. asperellum* M2RT4, *B. bassiana* ICIPE 706 and *H. lixii* F3ST1 as the most potent endophytic fungal isolates mediating improvement of tomato and nightshade anti-herbivore defense against *T. absoluta* through the reduction of adult oviposition, leafmining, pupation and adult emergence as compared to other treatments. *Trichoderma asperellum* M2RT4, *B. bassiana* ICIPE 706 and *H. lixii* F3ST1 could therefore be considered the best candidates for development of endophytic-based biopesticide and could be integrated as a component in a sustainable integrated *T. absoluta* management strategy for tomato and nightshade production systems. However, further studies are warranted to clearly understand the underlying mechanisms by which the presence of endophytic fungi within tomato and nightshade host plants affect *T. absoluta* as well as validate the findings under field conditions.

## Material and methods

### Fungal cultures

Fifteen fungal isolates belonging to five different genera (*Beauveria* (7), *Fusarium* (1), *Hypocrea* (1), *Metarhizium* (3) and *Trichoderma* (3)), obtained from the International Centre of Insect Physiology and Ecology (*icipe*)’s Arthropod Pathology Unit Germplasm, were used in this study (Table 1). These isolates were cultured on potato dextrose agar (PDA) (OXOID CM0139, Oxoid Ltd., Basingstoke, UK), except for *Metarhizium* which were cultured on Sabouraud dextrose agar (SDA) (OXOID CM0041, Oxoid Ltd., Basingstoke, UK), and maintained at 25 ± 2 °C in complete darkness. Conidia were harvested by scraping the surface of two to three-week-old sporulated cultures using a sterile spatula. The harvested conidia were then suspended in 10 mL sterile distilled water containing 0.05% Triton X-100 (MERCK KGaA, Darmstadt, Germany) and vortexed for 5 min at about 700 rpm to break conidial clumps and ensure a homogenous suspension^23,28^. Conidial concentrations were quantified using an improved Neubauer hemocytometer under a light microscope^72^. The conidial suspension was adjusted to a concentration of 1 × 10^8^ conidia mL^−1^ through serial dilution prior to inoculation of tomato and nightshade seeds.

**Table 1:** List of fungal isolates used in this study.

Fungal species	Isolate	Source	Origin	Year of isolation
*Beauveria bassiana*	ICIPE 35(4), ICIPE 35(6), ICIPE 35(12), ICIPE 35(15)	Coffee berry	Kenya	2009
*B. bassiana*	ICIPE 273	Soil	Mbita (Kenya)	2006
*B. bassiana*	ICIPE 706	Monocots	Kenya	2012
*Metarhizium anisopliae*	ICIPE 7	*Amblyomma variegatum*	Rusinga (Kenya)	1996
*M. anisopliae*	ICIPE 30	*Busseola fusca* (Lepidoptera)	Kenduba (Kenya)	1989
*M. anisopliae*	ICIPE 69	Soil	Matete (DRC)	1990
*Trichoderma* sp.	F2L41	Onion	Loitoktok (Kenya)	2012
*Trichoderma atroviride*	F2S21	Onion	Loitoktok (Kenya)	2012
*Trichoderma asperellum*	M2RT4	Maize and Sorghum	Nakuru (Kenya)	2009
*Fusarium proliferatum*	F2S51	Onion	Embu (Kenya)	2012
*Hypocrea lixii*	F3ST1	Maize and Sorghum	Nakuru, Embu and Kakamega (Kenya)	2009

Prior to commencement of the bioassays, spore viability was determined by plating evenly 0.1 mL of 3 × 10^6^ conidia mL^-1^ onto 9-cm Petri dishes containing SDA or PDA. Three sterile microscope cover slips (2 × 2 cm) were placed randomly on the surface of each inoculated plate. Plates were sealed with Parafilm and incubated in complete darkness at 25 ± 2 °C and were examined after 16–20 h. The percentage germination of conidia was determined from 100 randomly selected conidia on the surface area covered by each cover slip under a light microscope (×400) using the method described by Goettel and Inglis^72^. Conidia were considered to have germinated when the length of the germ tube was at least twice the diameter of the conidium^72^. Four replicates were used for each isolate.

### Seed inoculation and colonization assessment of endophyte isolates

Tomato (*Solanum lycopersicum* L. cv. "Moneymaker") and nightshade (*Solanum scabrum* Mill cv. "Giant nightshade") seeds (Simlaw Seeds Company Ltd., Nairobi, Kenya) were surface-sterilized by washing them up successively in 70% ethanol for 2 min followed by 1.5% sodium hypochlorite for three (3) min and finally rinsed three times in sterile distilled water. The surface sterilized seeds were placed on sterile filter paper on a clean working surface in a cabinet until the residual water evaporated. Effectiveness of the surface sterilization technique was confirmed by plating out 0.1 mL of the last rinse water onto potato dextrose agar and also imprinting of surface sterilized seeds onto PDA (tissue imprint) supplemented with 100 mg/L Streptomycin and plates were incubated at 25 °C for 14 days^73^. Seeds were then soaked overnight for 12 h in conidial suspensions titrated at 1 × 10^8^ conidia mL^−1^. For the controls, sterilized seeds were soaked overnight for 12 h in sterile distilled titrated (0.05% Triton X-100) water^23,28^. Seeds were then transferred into plastic pots (8 cm diameter × 7.5 cm high) containing the planting substrate with a volume of 0.5 L (mixture of manure and soil 1:5). The substrate was sterilized in an autoclave for 2 h at 121 °C and allowed to cool for 72 h prior to planting. Five seeds were sowed per pot and maintained at room temperature (25 ± 2 °C, 60% RH and 12:12 L:D photoperiod). Pots were transferred immediately after germination to the screen house (2.8 m length × 1.8 m width × 2.2 m height) at 25 ± 2 °C, 55% RH and 12:12 L:D photoperiod for 4–5 weeks. After germination, seedlings were thinned to two per pot and watered twice (~ 150 cm^3^) per day (morning and evening). No additional fertilizer was added to the planting substrate. Plants of 4–5 week-old were used for the various experiments.

To determine the colonization of inoculated fungal isolates in tomato and nightshade, plants were carefully uprooted from the pots 4–5 weeks after inoculation and washed under running tap water to remove any soil attached to the plants. Seedlings (ca. 30 cm in height) were divided into three different sections (ca. 5 cm long): leaves, stems and root sections using a sterile scalpel^23^. Five randomly selected leaf, stem and root sections from each plant were surface-sterilized as described above. The different plant parts were then aseptically cut under a laminar flow hood into 1 × 1 cm pieces before placing the pieces, 4 cm apart on PDA plates supplemented with a 0.05% solution of antibiotic (streptomycin sulphate salt)^23,28^. Plates were incubated at 25 ± 1 °C for 10 days, after which the presence of endophytes was determined. The last rinse water was also plated to assess the effectiveness of the surface sterilization procedure as described earlier. Plate imprinting was also conducted to assess effective surface sterilization of plant materials^74^. The colonization of the different plant parts was recorded by counting the number of pieces of the different plant parts that showed the presence of inoculated fungal growth/mycelia according to Koch’s postulates^75^. Only the presence of endophytes that were inoculated was scored. Fungal isolates were identified morphologically using slides which were prepared from the mother plates. Treatments were arranged in a randomized complete block design (RCBD) with four replicates per experiment^23^. The success rate of fungal endophyte colonization (%) of host plant parts was calculated as follows:

$${\text{Colonization }}\left( \% \right) = \frac{{\text{Number of pieces exhibiting fungal outgrowth}}}{{{\text{Total number of pieces plated out}} }} \times 100$$

### Insects

A colony of *T. absoluta* was established from wild adults and larvae collected from infested tomato leaves and fruits in Mwea (0° 36′ 31.3″ S 037° 22′ 29.7″ E), Kenya in June 2019. The moths were kept in ventilated, sleeved Perspex cages (40 × 40 × 45 cm) and were fed ad libitum with 10% honey solution placed to the top side of each cage as food source^76^. Four potted tomato plants were placed in the cages for oviposition. The plants were removed 24 h post-exposure to female insects and transferred to separate wooden cages (50 × 50 × 60 cm) ventilated with netting material at the sides and on the top until the eggs hatched. Leaves with larvae were removed from these plants, three days after the larvae hatched and placed into a clean sterile plastic containers (21 cm long × 15 cm wide × 8 cm high) lined with paper towel to absorb excess moisture and fine netting infused lid for ventilation. The larvae were supplied daily with fresh tomato leaves as food until they pupated. The pupae were collected from the plastic containers using a fine camel hair-brush and placed inside a clean plastic container for adult emergence. The colony was rejuvenated every three months through infusion, with infested tomato leaves collected from the field to reduce inbreeding^13,76^. Insects were maintained under a rearing condition of 28 ± 2 °C, 48% RH and 12:12 L:D photoperiod at the Animal Rearing and Quarantine Unit (ARQU) of *icipe* for five generations prior to bioassays^13^.

### Pathogenicity of endophytically-colonized tomato and nightshade plants on life history parameters of *Tuta absoluta*

Based on their ability to colonize plant tissues of both host plants, nine isolates (*B. bassiana* ICIPE 273, ICIPE 35(4), ICIPE 35(15), ICIPE 706, *F. proliferatum* F2S51, *T. harzianum* F2L41, *T. atroviride* F2S21, *H. lixii* F3ST1 and *T. asperellum* M2RT4) were tested for their impact against oviposition potential, eggs, larval and pupal mortality, adult emergence and longevity of *T. absoluta*. Two-day-old mated adults (10 individuals at sex ratio of 1:2 male: female) were exposed for 48 h to four-week-old endophytically-colonized host plant seedlings in Plexiglas cages (50 cm × 50 cm × 45 cm). Each cage contained four potted plants that represented a treatment, and was maintained at 25 ± 2 °C, 40% RH and 12:12 L: D photoperiod. All the treatments were arranged in a randomized complete block design and the experiment replicated four times. After 48 h post-exposure, insects were removed from the cages and introduced into clean cages (20 cm × 20 cm × 20 cm) and their survival was recorded by counting the number of live adults daily inside the cages until all moths died^23^. For each treatment, 10 female adults *T. absoluta* were monitored and the experiment was replicated four times.

Eggs that were laid on endophytically-colonized and control plants were maintained on the plants until they hatched. After hatching, larvae were allowed to feed upon their natal plants until they reached the 2nd and 3rd instars (approximately 8–10 d post-exposure). In the control, plants were not inoculated with fungal pathogens. For each treatment, the number of eggs laid on each plant was recorded as well as the number of mines and this was replicated four times. Using a fine paint brush, larvae were transferred into cages containing four potted plants that were in the same developmental stage as the one on which the caterpillars had hatched and had been feeding previously. Dead moths were placed on Petri dishes lined with damp sterilized filter paper to allow fungal growth on the surface of the cadaver (mycosis test). Caterpillars were allowed to feed freely on the potted plants in a cage until they pupated. For each treatment, pupation was recorded daily and pupae were collected from leaves 10–11 d post-exposure, counted and then incubated at 25 ± 2 °C. Adult emergence was determined for each treatment, and non-viable pupae were also counted. Following adult emergence from the endophytically-colonized and control plants, 20 adult moths were selected per treatment and the survival of F1 progenies was recorded daily until all moths died and this was replicated four times^77^. The moths were maintained in a cage as described in section “insects” above. A 10% honey solution was provided as food and cages maintained at 25 ± 2 °C, 48% RH and 12:12 L:D photoperiod. To confirm that the mortality of the moths was as a result of direct fungal infection, dead insects were placed on a moistened filter paper in Petri dishes and were observed for post-mortem fungal sporulation (mycosis test). Mycosis was assessed by surface sterilizing the dead moths with 1% sodium hypochlorite followed by three rinses with sterile distilled water, after which the sterilized cadavers were placed on sterile wet filter paper in sterile Petri dishes that were then sealed with Parafilm and kept at room temperature. Each treatment consisted of 10 insects and replicated four times.

### Statistical analyses

Colonization rate and count data (number of eggs, mines, pupae and adults) were tested for normality using Shapiro–Wilk test^78^ and homogeneity of variance using Levene test. The data were not normally distributed and variances were not homogeneous, therefore colonization rate and adult emergence data were analyzed with generalized linear model (GLM) using binomial distribution and logit link function. Count data were analyzed with generalized linear model (GLM) with negative binomial error distribution taking into account overdispersion. Whenever there was a difference, the means were separated using Tukey's honest significant difference (HSD) test using “agricolae” package in R^79^. The survival curves were generated using Kaplan–Meier estimator method, and log-rank test was used to compare the effect of the various fungal isolates on *T. absoluta* exposed adults and F1 progenies survival using the “Survival” package^80^. To test for differences in survival rate among the treatments, we calculated Cox’s proportional hazard^81^.

All analyses were performed using the R (version 3.6.2) statistical software packages^82^ and all statistical results were considered significant at the confidence interval of 95% (*P* < 0.05).

### Ethics approval

All insect rearing, handling and experiments were performed using standard operating procedures at the *icipe* Animal Rearing and Quarantine Unit as approved by the National Commission of Science, Technology and Innovations, Kenya (License No: NACOSTI/P/20/4253).

## Data Availability

The dataset generated during the current study are available from the corresponding author upon request.
